# A multicentered study on efficiency of noninvasive ventilation procedures(SAFE-NIV)

**DOI:** 10.3906/sag-2004-35

**Published:** 2021-06-28

**Authors:** Ebru Atike ONGUN, Oğuz DURSUN, Ayşe Berna ANIL, Ümit ALTUĞ, Özlem TEMEL KÖKSOY, Başak Nur AKYILDIZ, Serkan ÖZSOYLU, Tanıl KENDİRLİ, Serhan ÖZCAN, Rıza Dinçer YILDIZDAŞ, İlknur TOLUNAY, Bülent KARAPINAR, Mehmet Arda KILINÇ, Demet DEMİRKOL

**Affiliations:** 1 Division of Pediatric Critical Care, Department of Pediatrics, Faculty of Medicine, Akdeniz University Antalya Turkey; 2 Division of Pediatric Critical Care, Faculty of Medicine, Department of Pediatrics, İzmir Katip Celebi University and Tepecik Research and Training Hospital, İzmir Turkey; 3 Division of Pediatric Critical Care, Department of Pediatrics, Faculty of Medicine, Samsun Ondokuz Mayıs University, Samsun Turkey; 4 Division of Pediatric Critical Care, Department of Pediatrics, Faculty of Medicine, Erciyes University, Kayseri Turkey; 5 Division of Pediatric Critical Care, Department of Pediatrics, Faculty of Medicine, Ankara University, Ankara Turkey; 6 Division of Pediatric Critical Care, Department of Pediatrics, Faculty of Medicine, Çukurova University, Adana Turkey; 7 Division of Pediatric Critical Care, Department of Pediatrics, Faculty of Medicine, Ege University, İzmir Turkey; 8 Division of Pediatric Critical Care, Department of Pediatrics, Faculty of Medicine, İstanbul University, İstanbul Turkey

**Keywords:** HFNC, noninvasive positive pressure ventilation, children, respiratory failure

## Abstract

**Background/aim:**

To characterize the clinical course of noninvasive positive pressure ventilation (NIPPV) and high flow humidified nasal cannula ventilation (HFNC) procedures; perform risk analysis for ventilation failure.

**Material and methods:**

This prospective, multi-centered, observational study was conducted in 352 PICU admissions (1 month-18 years) between 2016 and 2017. SPSS-22 was used to assess clinical data, define thresholds for ventilation parameters and perform risk analysis.

**Results:**

Patient age, onset of disease, previous intubation and hypoxia influenced the choice of therapy mode: NIPPV was preferred in older children (p = 0.002) with longer intubation (p < 0.001), ARDS (p = 0.001), lower respiratory tract infections (p < 0.001), chronic respiratory disease, (p = 0.005), malignancy (p = 0.048) and immune deficiency (p = 0.026). The failure rate was 13.4%. sepsis, ARDS, prolonged intubation, and use of nasal masks were associated with NIV failure (p = 0.001, p < 0.001, p < 0.001, p = 0.025). The call of intubation or re-intubation was given due to respiratory failure in twenty-seven (57.5%), hemodynamic instability in eight (17%), bulbar dysfunction or aspiration in 5 (10.6%), neurological deterioration in 4 (8.5%) and developing ARDS in 3 (6.4%) children. A reduction of less than 10% in the respiration within an hour increased the odds of failure by 9.841 times (OR: 9.841, 95% CI: 2.0021–48.3742). FiO2 > 55% at 6th hours and PRISM-3 >8 were other failure predictors. Of the 9.9% complication rate, the most common complication was pressure ulcerations (4.8%) and mainly observed when using full-face masks (p = 0.047). Fifteen (4.3%) patients died of miscellaneous causes. Tracheostomy cannulation was performed on 16 children due to prolonged mechanical ventilation (8% in NIPPV, 2.6% in HFNC)

**Conclusion:**

Absence of reduction in the respiration rate within an hour, FiO2 requirement >55% at 6th hours and PRISM-3 score >8 predict NIV failure.

## 1. Introduction

The introduction of new ventilation strategies at pediatric intensive care (PICU) units broadened horizons from conventional mechanical ventilation (MV) to noninvasive ventilation (NIV) for children who require advanced respiratory support. This alternative strategy improves respiratory functions in children with acute respiratory failure (ARF) by preserving intact airway reflexes while avoiding the potential risks of endotracheal intubation [1]. Facilitating early liberation from intubation is another advantage especially for those delivering MV [2]. NIV improves oxygenation and effective ventilation by decreasing the work of breathing and unloading respiratory muscles. The outcome is better oxygen delivery with enhanced gas exchange and better ventilation/perfusion ratio in patients with hypoxic and/or hypercapnic ARF [3,4]. The efficiency has been studied in several clinical conditions including acute pneumonia [5], bronchiolitis [6], status asthmaticus [7] and post-extubation respiratory failure [8]. Physiological studies also confirm the positive influence on respiratory functions [9,10].

Noninvasive positive pressure ventilation (NIPPV) techniques include continuous positive airway pressure (CPAP) and bilevel positive airway pressure (BIPAP). They are used in various etiologies [11–13] and the decision of modality depends on the nature of respiratory failure: CPAP for hypoxic ARF and smaller children, BIPAP for older children with hypoxic and/or hypercapnic ARF [14]. Although not classified as positive pressure ventilation, high flow humidified nasal cannula ventilation (HFNC) is gaining interest in pediatric practice and is presented as an alternative to CPAP in infants. HFNC enhances oxygenation by providing anatomic oxygen reservoirs and washing out the dead airway space [14]. It drives a CPAP-like effect at 6 L/min by generating a positive pressure through the respiratory cycle [14,15]. 

NIV success relies upon appropriate patient selection, choice of modality and experienced trained staff [14]. In contrary to adult consensus conference statements [2,16], the guideline addressing the clinical applications (criteria for initiation and/or discontinuation) in children remain limited in this manner [17]. The decision of failure is mostly left to the discretion of the treating physician. Moreover, there is a varying range of discrepancy in inter-unit ventilation strategies between health-care facilities [17]. This prospective, multicenter, observational study was planned to characterize the clinical course of NIPPV and HFNC implementations and observe success rates in decreasing the necessity of mechanical ventilation or re-intubation. The secondary outcome is to assess risk analysis for respiratory failure and examine therapy-attributed complications (if any).

## 2. Materials and methods

The study was conducted with the collaboration of eight PICUs (universities or teaching hospitals governed by the ministry of health) all across the country between December 2016 and December 2017. The approval of the local ethical committee was obtained from Akdeniz University Faculty of Medicine, Clinical Research Ethics Committee (no: 70904504/525, date: 11.18.2016). The study was also registered at clinicaltrials.gov (approval number: 70904504/525). All critically-ill children from 1 month to 18 years requiring NIPPV or HFNC in the intensive care settings were enrolled in this study. We did not set any criteria for NIPPV or HFNC institution, therapy discontinuation and the call of intubation. The clinical decisions were led by the discretion of the attending senior physician at each center.          

A three-page case report form (CRF) was designed to collect data *.* The patient demographics were grouped according to the patient age (infants less than 12 months, toddlers/preschool-aged children between 13 and 60 months, school-age/adolescents between 61 months to 18 years) and the underlying disease (including acute and chronic settings). We categorized acute disease as i) lower respiratory tract infections (LRTI) ii) conditions related to bronchospasm including bronchiolitis and asthma attacks, iii) upper respiratory tract infection (URTI): laryngitis and laryngotracheobronchitis (croup), iv) clinically-proven heart failure, v) central nervous system (CNS) related disease: any disorder resulting in an incapability to sustain airway maneuvers, vi) sepsis, vii) ARDS defined by the Pediatric Acute Lung Injury Consensus Conference Group (PALICC) [18], and finally vii) postoperative laryngeal stridor. The underlying chronic conditions were classified by Feudtner’s coding system [19]. The second page has consisted of a multi-colon table to record the respiratory rate (RR), heart rate (HR), systemic, mean and diastolic blood pressures (BP), the pediatric risk of mortality-3 score (PRISM-3) and pediatric logistic organ dysfunction score (PELOD), Glasgow coma scale (GCS), comfort score, and blood gas parameters. The device mode and settings, types of masks used and drugs used for sedation (if given) were obtained. We adjusted RR nd HR for age above the 99th percentile to determine the abnormality above normal limits to define tachypnea and tachycardia [20]. Data were collected up to 5 days: immediately before commencing respiratory support, at 1st, 2nd, 6th, 24th, 48th hours, then daily. However, the statistical analysis regarding NIV failure involved the first 48 h. The clinical outcome including the cause of intubation or re-intubation (if so the timing of intubation), the complications, the length of ventilation therapy, PICU stay and hospitalization were also recorded.

    Children were divided into two main groups whether if they achieved therapy success or not. Therapy failure was defined as the necessity of endotracheal intubation or re-intubation within the study period. In case of a failed trial, the collaborators were asked to define the cause of failure as patient-ventilator asynchrony, use of misfit interface, excessive air leaks, neurological deterioration (GCS < 8 or deterioration over three units), risk of aspiration or bulbar dysfunction (loss of swallowing), hemodynamic alterations, presence of arrhythmia, pneumothorax, and progression to ARF (worsening respiratory condition). The definition of ARF was adapted from the criteria of Teaque as an unsustainable alveolar exchange to meet the metabolic cellular demands [12]. CRF has also consisted of several failure risk factors previously defined in the literature, to set up criteria to anticipate ARF such as PaO2/FiO2 < 200 [5,21] or FiO2 necessity over 80% at 1st hour [22], blood gas pH < 7.25 after 2 h [23], failure to achieve a decrease in RR within 6 h, clinically observed increased work of breathing (respiratory score of 1–2 by Monachan’s pediatric early warning system) [24], progression to moderate/severe ARDS (oxygenation index > 8) [18].

 Subgroup analyses were performed depending on the mode of therapy and the therapy implementation (first-line therapy or rescue therapy). HFNC failures consisted of children who delivered HFNC as respiratory support throughout the study and encountered intubation or re-intubation. 

### 2.1. Statistical analysis

IBM SPSS Statistics for Windows, v. 22.0 (IBM Corp., Armonk, NY, USA) was used for statistics. Pearson chi-square and Fisher’s exact test were used to assessing categorical data; Mann–Whitney U test and Student’s t-test for nonnormally and normally distributed continuous variables (after performing Shapiro–Wilks test to control normality assumptions) We used repeated-measures ANOVA with posthoc Bonferroni test to analyze the change in the means of continuous variables at different time points. The Friedman test alongise the Bonferroni correction were used for nonparametric comparison of parameters measured at different times. The receiver operating characteristic (ROC) curve analysis was applied to identify the cut-off points of continuous variables with significant differences between groups and the Youden index to determine the optimal cutoff point of each variable of interest. Continuous variables were then classified into positive or negative groups according to the cutoff point. We then, performed a multivariate logistic regression analysis to determine independent risk factors associated with therapy failure. Data are expressed as n (%), mean ± standard deviation (SD), or median (minimum-maximum). P-value < 0.05 was considered statistically significant.

## 3. Results

### 3.1. Patient characteristics

A total of 352 children of male gender predominance (56.8%) with a median age of 18 months (1.5–204 months) and the bodyweight of 10 kg (min-max: 2.1–120 kg) was enrolled in the study. The distribution of age category was infanthood in 152 patients (43.2%), toddlers/preschool era in 119 (33.8%) patients and school-age/adolescents in 81 (23%) patients. LRTI and hypoxic ARF were the common causes of NIV administration (47.4%, 67.9%; Table 1). Overall, 71.6% of the population had an underlying chronic condition involving (in decreasing order) cardiac disease, neurological conditions, and immune deficiency (25.6%, 21.9%, 7.1%; Table 2). The median duration of NIV administration was 72 h (min-max: 36–120 hours). 

**Table 1:** General demographics of the study population

Variable	N = 352	Variable	N = 352
Acute Respiratory Failure (ARF)			
Hypoxic ARF	239 (67.9%)	Hypercapnic ARF	113 (32.1%)
Mode of therapy, n (%)			
NIPPV	125 (35.5%)	HFNC	227 (64.5%)
Therapy implementation, n (%)			
First-line therapy	159 (45.2%)	Rescue therapy	193 (54.8%)
ICU Scores			
PRISM-3 score*	5 (2-51)	GCS**	13.76 ± 1.67
PELOD score*	6 (2-50)	Comfort score**	23.06 ± 3.65
Sedoanalgesia (n=165, 46.9%)			
Dexmedetomidine	53/165 (32.1%)	Fentanyl	6/165 (3.6%)
Ketamine	50/165 (30.3%)	Paracetamol	2/165 (1.2%)
Midazolam	50/165 (30.3%)	Morphine	2 (1.2%)
Chloralhydrate	4/165 (2.4%)		
Length of stay (days)			
Intensive care*	8 (2-98)	Hospitalization*	14 (4-98)

**Table 2:** Acute and Chronic Onset of Diseases

Acute onset of disease		The underlying chronic conditions	
LRTI	167 (47.4%)	Cardiac disease a	90 (25.6%)
Bronchiolitis/asthma attack	61 (17.3%)	Neurological conditions	77 (21.9%)
URTI	12 (3.4%)	Immune deficiency	25 (7.1%)
Postoperative stridor	12 (3.4%)	Metabolic disease	22 (6.3%)
Heart failure	41 (11.6%)	Respiratory disease b	20 (5.7%)
Sepsis	30 (8.5%)	Genetic	19 (5.4%)
CNS-related	21 (6%)	Genitourinary	16 (4.5%)
ARDS	13 (3.7%)	Malignancy	11 (3.1%)
		Gastrointestinal disease c	9 (2.6%)

### 3.2. Decision of modality and device selection  

Table 3 presents the population demographics and outcome measures according to treatment modality. Both NIPPV and HFNC were used as first-line or rescue therapy (rescue therapy: 48.8% versus 58.1%, p = 0.092). Several factors acted upon the selection of ventilation mode such as, patient age, duration of previous intubation, the nature of acute and chronic disease. NIPPV was the preferred modality in older children with longer intubation periods (p = 0.002, p < 0.001), in an acute of setting of ARDS and LRTI (p = 0.001, p < 0.001) and in an underlying condition such as chronic respiratory disease, malignancy and immune deficiency (p = 0.005, p = 0.048, p = 0.026). The choice of method were based on the degree of age-adjusted tachypnea (NIPPV: 65.3%, HFNC: 52.9%, p = 0.022) and SpO2/FiO2 values (NIPPV: 175.24 ± 52.99, HFNC: 192.83 ± 55.23; p = 0.005), instead of blood gas pCO2 (NIPPV: 45.39 ± 11.01 mmHg, HFNC: 43.55 ± 9.54 mmHg, p = 0.144).

**Table 3:** The descriptive analysis of NIV modalities (HFNC–NIPPV).

	NIPPV	HFNC	o		NIPPV	HFNC	p
Age (months)*	27 (1.5–204)	14 (1.5–192)	0.002	Bodyweight (kg)*	10.5 (3–120)	9 (2.1–100)	0.004
Infanthood	46 (36.8%)	106 (46.7%)		NIV duration (hours)*	72 (6–480)	52 (6–48024)	0.012
Toddlers/preschool age	36 (28.8%)	83 (36.6%)	0.001	Previous intubation (hours)*	140 (28–726)	48 (25–1000)	< 0.001
School age/adolescents	43 (34.4%)	38 (16.7%)		PRISM-3 score*	6 (2–38)	5 (2–51)	0.264
Male gender, (n)	72 (57.6%)	128 (56.4%)	0.826	PELOD score*	6 (2–31)	8 (2–50)	0.240
Acute onset of disease				Underlying chronic conditions			
LRTI	76 (60.8%)	91 (40.1%)	< 0.001	Cardiac disease	19 (15.2%)	71 (31.1%)	0.001
Bronchiolitis/asthma	43 (34.4%)	108 (47.6 %)	0.017	Neurological condition	33 (26.4%)	44 (19.4%)	0.128
URTI	-	11 (4.8%)	0.012	Metabolic disease	12 (9.6%)	10 (4.4%)	0.054
Postoperative stridor	4 (3.2%)	8 (3.5%)	0.569	Genetic disease	6 (4.8%)	13 (5.7%)	0.713
ARDS	10 (8%)	3 (1.3%)	0.001	Respiratory disease	13 (10.4%)	7 (3.1%)	0.005
Heart failure	10 (8%)	31 (13.7%)	0.113	Genitourinary disease	6 (4.8%)	10 (4.4%)	0.865
Sepsis	10 (8.8%)	19 (8.4%)	0.894	Gastrointestinal disease	3 (2.4%)	6 (2.6%)	0.896
CNS-related	5 (4%)	16 (7%)	0.248	Malignancy	7 (5.6%)	4 (1.8%)	0.048
				Immune deficiency	14 (11.2%)	11 (4.8%)	0.026
Outcome							
PICU stay (days)*	11 (2–98)	7 (2–69)	< 0.001	Hospital discharge (days)*	20 (5–98)	12 (4–95)	< 0.001
Tracheostomy (n)	10 (8%)	6 (2.6%)	0.021	Exitus, (n)	8 (6.4%)	7 (3.1%)	0.140

The children delivering NIPPV were more likely to receive treatment via specific noninvasive ventilation devices (93.6%) with full-face masks (48%), nasal masks (19.2%), and oronasal masks (11.2%). 

### 3.3. Sedation procedures and complications

To optimize care, 46.9% of children received a sedation procedure under assessment of comfort scores (Table 1). The most frequent drugs were dexmedetomidine, ketamine, and midazolam administered at intermittent bolus doses (32.1%, 30.3%, and 30.3% respectively). Sedation sessions were applied to children using (in decreasing order), full-face masks 55%, nasal masks 45.8%, nasal prong 45.3%, and oronasal masks 42.9%. 

A total of 35 (9.9%) complications were observed regardless of therapy mode (NIPPV: 14.4% vs. HFNC: 7.5%; p = 0.063). The incidence varied between different mask types: 25% in nasal masks, 11.7% in full-face masks, 8.3% in nasal prongs and 7.1% in oronasal masks. The most common complication was pressure ulcerations (skin/mucosa breakdown) (17/352, 4.8%). The ones who failed their NIV sessions had increased complication rates (p < 0.001). 

### 3.4. Therapy failure and risk analysis 

Forty-seven (13.4%) children were marked as NIV failures and the majority had to be intubated within 48 h (in 4 children, the decision of failure extended beyond 48 h: one in NIPPV and 3 in HFNC). Instead of the patient characteristics (age, sex), the nature of acute respiratory failure (hypoxic or hypercapnic), NIV modality (NIPPV/HFNC) or the therapy institution (first-line/rescue therapy), the acute onset of disease (sepsis and ARDS: p = 0.001, p < 0.001) and the underlying chronic conditions (metabolic and genitourinary problems: p = 0.017, p = 0.048) were related to NIV failure (Table 4). These patients had longer intubation period prior to NIV administration, higher PRISM-3, PELOD scores and lower GCS (p < 0.001, p < 0.001, p < 0.001, p = 0.003, respectively). The call of intubation/re-intubation was given due to respiratory failure in 27 (57.5%) children, hemodynamic instability in 8 (17%), bulbar dysfunction or aspiration in 5 (10.6%) children, neurological deterioration in 4 (8.5%) children and developing ARDS in 3 (6.4%) children. The use of a nasal mask during NIPPV was also associated with NIV failure (15.2% versus 5.6%; p = 0.025).  

**Table 4:** The descriptive analysis of NIV failure.

Variable	failure	NIVsuccess	p	Variable	NIVfailure	NIVsuccess	p
Age (months)*	36 (2–192)	16 (1.5–204)	0.137	PRISM-3 score*	13 (2–51)	5 (2–36)	<0.001
Bodyweight (kg)*	12 (2.1–60)	9.7 (2.7–120)	0.453	PELOD score*	14 (2–50)	6 (2–44)	<0.001
Male gender	28 (59.6%)	172 (56.4%)	0.682	GCS (mean ± SD)	13.21 ± 1.67	13.85 ± 1.66	0.003
Hypoxic ARF	34 (72.3%)	205 (67.2%)	0.483	Complications	15 (31.9%)	20 (6.6%)	<0.001
Acute onset of disease				Underlying chronic disease			
LRTI	25 (53.2%)	142 (46.6%)	0.397	Cardiac	4 (8.5%)	86 (28.2%)	0.004
Bronchiolitis/asthma	1 (2.1%)	60 (19.7%)	0.003	Neuromuscular	15 (31.9%)	62 (20.3%)	0.074
URTI	-	11 (3.6%)	NA	Metabolic	7 (14.9%)	15 (4.9%)	0.017
ARDS	6 (12.8%)	7 (2.3%)	<0.001	Genetic	3 (6.4%)	16 (5.2%)	0.478
Heart failure	4 (8.5%)	37 (12.1%)	0.471	Respiratory	3 (6.4%)	17 (5.6%)	0.515
Sepsis	11 (23.4%)	19 (6.2%)	0.001	Genitourinary	5 (10.6%)	11 (3.6%)	0.048
CNS-related	5 (10.6%)	16 (5.2%)	0.132	Gastrointestinal	3 (6.4%)	6 (2.0%)	0.105
Postoperative stridor	-	12 (3.9%)	NA	Malignancy	3 (6.4%)	8 (2.6%)	0.170
				Immune deficiency	5 (10.6%)	20 (6.6%)	0.229
Therapy mode				Therapy implementation			
NIPPV	22 (46.8%)	103 (33.8%)	0.082	First-line therapy	22 (46.8%)	137 (44.9%)	0.808
HFNC	25 (53.2%)	202 (66.2%)		Rescue therapy	25 (53.2%)	168 (55.1%)	
Device selection				Interfaces			
Specific NIPPV device	18 (38.3%)	98 (32.1%)	0.072	Nasal mask	7 (15.2%)	17 (5.6%)	0.025
MV with NIV mode	3 (6.4%)	5 (1.6%)		Oronasal mask	3 (6.5%)	11 (3.6%)	0.408
Specific HFNC device	26 (55.3%)	202 (66.2%)		Full-face mask	11 (23.9%)	50 (16.4%)	0.210
				Nasal Prong	27 (57.4%)	227 (74.4%)	0.016
Signs of respiratory failure							
Tachypnea**	33 (70.2%)	167 (55.1%)	0.052	Bloodgas pH <7.25 (2nd hour)	2 (4.3%)	4 (1.3%)	0.186
SpO2/FiO2 < 200 (1st hour)	38 (82.6%)	148 (49.3%)	< 0.001	RR decline >10% (1st hour)	44 (93.6%)	178 (58.9%)	<0.001
FiO2 > 80% (1st hour)	14 (37.8%)	27 (9.6%)	< 0.001	RR decline >10% (6th hour)	46 (97.9%)	77 (25.5%)	<0.001
The length of stay (days)							
PICU stay*	26 (4–98)	7 (2–87)	< 0.001	Hospitalization*	32 (4–98)	13 (5–98)	<0.001

**Figure 1:**
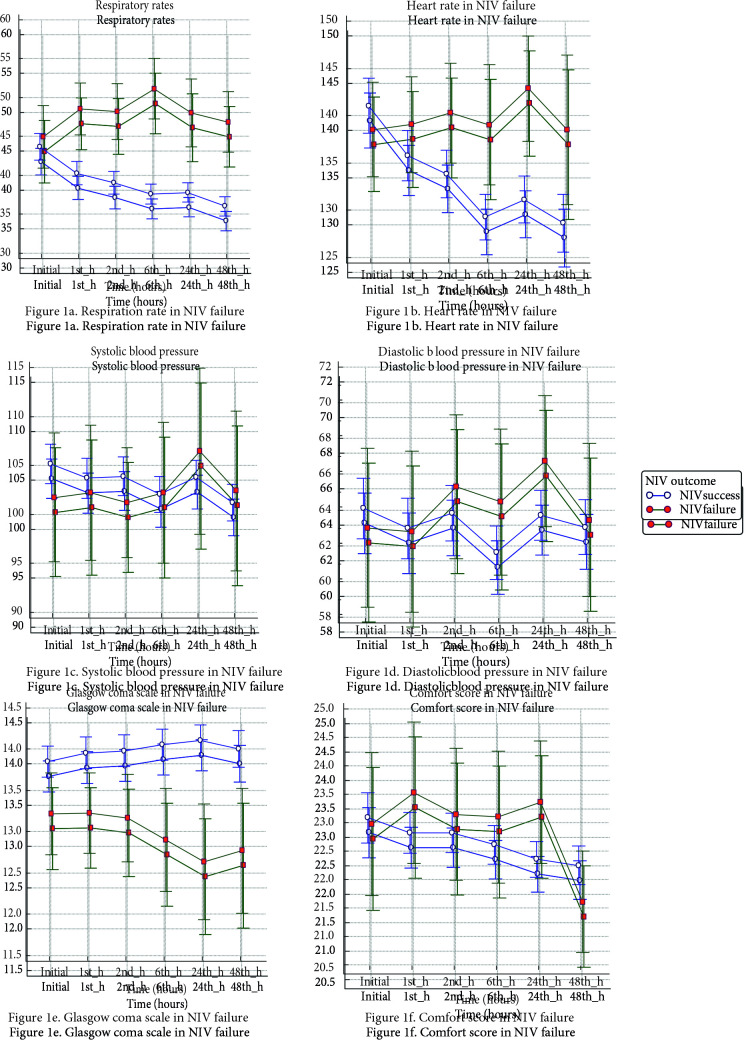
a) Respiration rate in NIV failure; b) Heart rate in NIV failure; c) Systolic blood pressure in NIV failure; 1d) Diastolic blood pressure in NIV failure; 1e) Glasgow coma scale in NIV failure; 1f) Comfort score in NIV failure.

**Figure 2:**
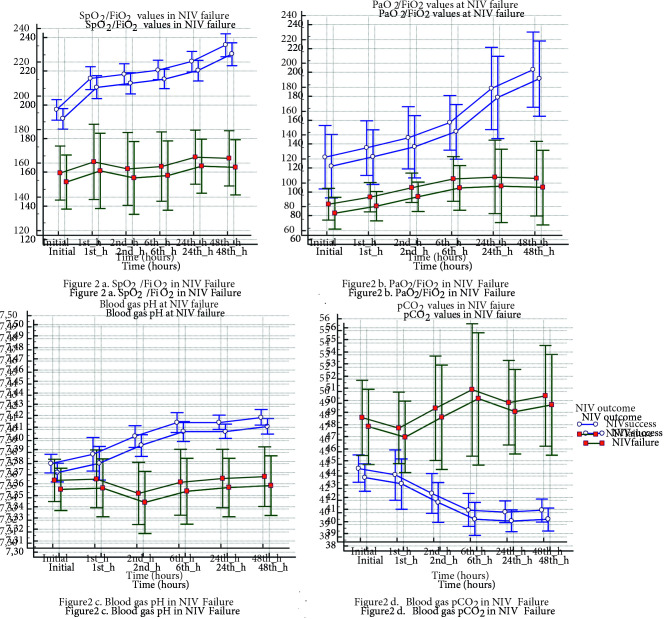
a) SpO2/FiO2 in NIV failure; 2b) PaO2/FiO2 in NIV failure; 2c) Blood gas pH in NIV failure; 2d) Blood gas pCO2 in NIV failure

Figures 1 and 2 present the vital parameters and blood gas analysis in NIV failure (Figure 1a: Respiration rate, Figure 1b: Heart rate, Figure 1c: Systolic blood pressure, Figure 1d: Diastolic blood pressure, Figure 1e: Glasgow coma scale, Figure 1f: Comfort score; Figure 2a: SpO2/FiO2, Figure 2b: PaO2/FiO2, Figure 2c: Blood gas pH, Figure 2d: Blood gas pCO2 in NIV Failure). NIPPV failures displayed significant device settings: higher inspiratory pressure (IPAP) requirement with lower tidal volumes at all times (Table 5). On the contrary, the initial settings of flow-rates in HFNC remained more or less the same regardless of therapy success (p = 0.973).

**Table 5:** The device settings in NIV failure.

	Therapy failure		Therapy success	
Variables	Time	Mean ± SD	Mean ± SD	p
NIPPV Therapy				
	Time	Mean ± SD	Mean ± SD	
IPAP	Initial	15.63 ± 4.73	12.77 ± 4.19	0.013
	1. hour	15.88 ± 4.99	12.38 ± 3.21	0.006
	2. hour	17.01± 5.26	12.93 ± 4.06	0.002
	6. hour	16.44 ± 5.44	13.03 ± 4.03	0.015
	24. hour	17.01 ± 5.82	12.88 ± 4.12	0.007
	48. hour	18.80 ± 5.29	12.64 ± 4.48	0.001
p	< 0.001		0.200	
EPAP /PEEP	Initial	5.58 ± 0.84	5.57 ± 1.07	0.923
	1. hour	5.61 ± 0.78	5.57 ± 0.99	0.705
	2. hour	5.71 ± 1.21	5.62 ± 1.07	0.913
	6. hour	5.59 ± 0.87	5.68 ± 1.09	0.993
	24. hour	5.80 ± 1.08	5.67 ± 1.15	0.555
	48. hour	5.82 ± 0.87	5.77 ± 1.15	0.676
p	0.999		0.853	
FiO2 (%)	Initial	68.89 ± 22.20	55.98 ± 17.76	< 0.001
	1. hour	64.12 ± 18.05	50.54 ± 11.21	< 0.001
	2. hour	56.25 ± 13.60	49.46 ± 9.91	< 0.001
	6. hour	59.41 ± 14.78	49.13 ± 9.45	< 0.001
	24. hour	58.93 ± 16.66	48.35 ± 9.72	< 0.001
	48. hour	56.50 ± 7.47	45.06 ± 8.24	< 0.001
p	0.257		<0.001	
Inspiration time	Initial	0.66 ± 0.24	0.64 ± 0.22	0.819
	1. hour	0.66 ± 0.25	0.65 ± 0.22	0.906
	2. hour	0.68 ± 0.25	0.65 ± 0.22	0.722
	6. hour	0.63 ± 0.20	0.64 ± 0.22	0.852
	24. hour	0.62 ± 0.19	0.66 ± 0.21	0.551
	48. hour	0.69 ± 0.18	0.67 ± 0.21	0.814
p	0.999		0.416	
Rise time	Initial	2.68 ± 1.47	2.51 ± 1.45	0.801
	1. hour	2.68 ± 1.47	2.57 ± 1.54	0.954
	2. hour	2.65 ± 1.53	2.56 ± 1.52	0.961
	6. hour	2.78 ± 1.53	2.57 ± 1.51	0.792
	24. hour	2.73 ± 1.63	2.54 ± 1.54	0.873
	48. hour	2.1 ± 1.28	2.37 ± 1.56	0.752
p	0.999		0.416	
Tidal volume(mL/kg)	Initial	6.76 ± 1.86	7.81 ± 1.81	0.049
	1. hour	7.04 ± 1.69	7.83 ± 1.88	0.138
	2. hour	6.62 ± 1.69	7.81 ± 1.72	0.025
	6. hour	6.59 ± 1.67	7.81 ± 1.64	0.017
	24. hour	6.74 ± 1.28	7.85 ± 1.64	0.034
	48. hour	7.05 ± 1.55	7.74 ± 1.37	0.387
p	0.448		0.752	
Leakage	Initial	20.31 ± 12.21	29.88 ± 18.83	0.076
	1. hour	20.31 ± 12.04	27.9 ± 17.69	0.200
	2. hour	22.07 ± 12.64	30.12 ± 18.82	0.194
	6. hour	23.43 ± 14.52	29.26 ± 18.93	0.478
	24. hour	27.42 ± 16.75	28.61 ± 18.44	0.927
	48. hour	22.63 ± 13.42	29.83 ± 19.59	0.350
p	0.413		0.570	
Mandatory rate	Initial	23.44 ± 5.40	22.45 ± 6.40	0.677
	1. hour	24.33 ± 7.30	22.0 ± 5.46	0.502
	2. hour	22.36 ± 4.94	21.54 ± 5.26	0.727
	6. hour	25.31 ± 8.01	21.3 ± 4.78	0.146
	24. hour	24.55 ± 9.06	20.8 ± 4.55	0.355
	48. hour	24 ± 8.07	20.7 ± 4.51	0.350
p	0.304		0.283	
HFNC therapy				
Flow rate	Initial	17.27 ± 7.83	17.29 ±7.80	0.973
	1. hour	18.42 ± 8.71	17.79 ± 7.78	0.739
	2. hour	18.01 ± 7.08	17.68 ± 7.62	0.529
	6. hour	19.67 ± 8.71	17.03 ± 7.59	0.085
	24. hour	20.90 ± 9.62	15.64 ± 7.12	0.008
	48. hour	20.88 ± 10.11	14.39 ± 7.43	0.007
p	0.027		< 0.001	
FiO2 (%)	Initial	67.63 ± 18.59	50.73 ± 14.42	< 0.001
	1. hour	68.75 ± 19.96	49.88 ± 14	< 0.001
	2. hour	67.35 ± 18.72	48.64 ± 12.94	< 0.001
	6. hour	68.01 ± 18.59	47.78 ± 12.15	< 0.001
	24. hour	60.77 ± 20.9	43.36 ± 10.99	0.002
	48. hour	62.78 ± 19.54	41.29 ± 10.54	< 0.001
p	0.182		< 0.001	

ROC curve analysis for NIV failure has indicated the cutoff values (calculated by the Youden index) for FiO2 >55% at the 6th hour and PRISM-3 score >8 (AUC: 0.762, 95% CI: 0.7100–0.809, sensitivity 89.87%, specificity 70.79% and AUC: 0.756, 95% CI:0.695–0.810, sensitivity: 86%, specificity: 77.2%). A multivariate logistic regression was launched to define the deteriorating child at risk for NIV failure and included the variables such as respiration rate, FiO2 at the 6th hour, PRISM-3 score, ARDS, sepsis, and metabolic andgenitourinary disease. The model identified a less than 10% decrease in respiration in the 1st hour (OR: 9.841, 95% CI: 2.0021–48.3742, p < 0.004), FiO2 nesessity >55% at 6th hours (p = 0.002, OR: 5.2936 95% CI: 1.7964–15.5995, p = 0.002) and PRISM-3 score >8 as independent risk factors for NIV failure OR: 3.9011 95% CI: 1.3370–11.3827, p = 0.012).   

### 3.5. Prognosis

Of forty-seven (13.4%) ventilation failures, 15 (4.3%) children died of miscellaneous causes (time of death: median 15.5 days, min-max: 4–32 days). Tracheostomy cannulation was performed on 16 children due to prolonged invasive mechanical ventilation (8% in NIPPV, 2.6% in HFNC)

## 4. Discussion 

There were several outcomes in the current study. The immediate effect of noninvasive ventilation on patients’ respiration was observed at the very early stages of therapy: the successful trials were able to lower down their RR at least 10% within an hour. The changes in ventilation parameters appeared to be a significant indicator to observe therapy response [25]. In a previous study from Bakalli et al., children achieved success with a positive predictive value of 88.2% (95% CI: 72.5%–96.6%), when they were able to reduce their RR more than 10 respiration/min in 2 h [25]. Similarly, Mayordomo-Colunga et al. stated the absence of RR decline at 1st and 6th hours in NIPPV failure [26]. Several models also predict HFNC failure such as RR above the 90th percentile for age at triage [1,27], no RR or HR change within 60 min [28] and worsening pediatric early warning scores [29]. The failure rate (13.4%) in this study was within the reported ranges of 3% and 30% [3,8,30]. Neither the treatment modality (NIPPV/HFNC) nor the therapy institution (first-line/rescue therapy) was associated with NIV failure. Literature data suggest that longer intubation [3], higher FiO2 requirement [3,5,31], PRISM-3 and PELOD scores [31], hypoxic ARF, parenchymal lung disease and ARDS [3,5,25,32] are recognized as potential risk factors. However all of the published researches report different cutoff values other than thresholds obtained in the current study (PRISM-3 score > 8 and FiO2 requirement > 55 at 6 hours). Our findings were lower than previous data: a cut-off level of 10 for PRISM-3 in Bakalli’s research [25] or FiO2 > 80% in the first hour [22], FiO2 > 57% [5], and FiO2 > 60% [25]. 

Unlike literature data reporting pneumonia as the sole factor for NIV failure [2,3], our findings pointed out sepsis and ARDS. The impact of sepsis is a little-known subject (mostly studied in malignancies) in the acute setting of NIV [33–36]. ARDS, on the other hand, is a well-defined challenge for therapy success [31]. In 2015, the PALICC group stated the beneficiary effects of noninvasive ventilation especially for an immune-compromised child under the condition of close monitoring and presence of trained healthcare providers. However, the recommendations were limited to the early ARDS stages; they concluded not to delay intubation in case of severe disease or no clinical improvement [18]. The current study’s ARDS rates were relatively lower: 8% in NIPPV, 1.3% in HFNC sessions and approximately half of the patients had to be intubated following the NIV-course (46.2%, 6/13). Yet, our findings could not be attributed to true incidence of ARDS due to the study’s observational design. Further, larger scaled investigations are needed to focus on ARDS and noninvasive ventilation. 

Distinct characteristics regarding device settings emerged from this study. Despite a steady IPAP increase, we noted steady end-expiratory pressure (EPAP/PEEP) levels in children receiving NIPPV even if they demanded higher oxygen to improve work of breathing. EPAP and PEEP are two synonymous parameters known to minimize alveolar collapse and improve oxygenation [4]. Apparently, the choice of intervention favored endotracheal in hypoxic patients instead of elevating EPAP/PEEP elevation in hypoxic patients. We think this outcome might be the reflection of PALICC recommendations on ARDS ‘not delaying intubation in the absence of clinical improvement’ [18].

Age, length of previous intubation, underlying conditions (acute and chronic settings) were noted to play a role in the decision of ventilation modality. HFNC was the preferred method of NIV in smaller children acquiring upper respiratory system problems and bronchiolitis/asthma attacks. NIPPV, on the other hand, was mostly used in older children with prolonged intubations, primary parenchymal lung disease (pneumonia, ARDS, chronic lung disease), malignancy, and immune deficiency. Complex chronic conditions inevitably influence the ventilation strategies [8,19,33]. SCARF (early CPAP in acute respiratory failure) study is a recent, randomized, controlled study performed in children with a high-risk combination of impaired immunity and ARF [37]. Given the evidence from SCARF study, NIPPV remained as a reserved ventilation method for more distressed population with malignancy or immune deficiency who possess greater risks for endotracheal intubation. Our findings shared a common ground with the SCARF study: NIPPV was chosen for more tachypneic and hypoxic (lower SpO2/FiO2) children who required higher FiO2 and longer therapy sessions in the study centers. Of note, children receiving NIPPV were more likely to require tracheostomy cannulation when they were intubated or re-intubated, even their initial PRISM-3 or PELOD scores were similar. This outcome verified the fact that, the nature of underlying problem were more important when compared to disease severity. The findings were also supported by two previous investigations that demonstrated any insignificance between PIM-2 scores and ventilation failure [22,33].   

NIPPV implementations confront certain challenges amongst the youth such as air leakages, higher respiration rates, and reduced respiratory efforts to achieve patient-device synchrony. Therefore, the success depends on the appropriate device selection, including interface, circuit, device, and device settings [12]. Our local practices favored the application of specific noninvasive ventilator devices (capable of compensating high leakage) with the use of full-face masks. Recent advances in the manufacturing industry have resulted in different-sized masks, which enable more applicable, patient-friendly utilization at all ages [8]. Full-face masks provide both comfort (by spreading the mask-fit pressure over a larger surface beyond the nose) and fewer pressure-related complications than oronasal masks [12]. Besides, the efficiency are shown in terms of breathing pattern and gas exchange as much as the oronasal masks [12]. The practice regarding the selection of interfaces in the current study was consistent with the PALICC group who have suggested the application of full-face or oronasal masks in pediatric ARDS for effective ventilation [18].

Several studies emphasized the impact of nasal masks [12,38]. These masks are noted to have several advantages such as more comforting patient-friendly use, fewer gastric distension, better feeding tolerance, safer for aspiration [12], and lower risks of NIV failure [38]. Opposing this hypothesis, we observed significant complication and failure rates with the use of nasal masks. One possible explanation might be the mouth breathing of the youngster as a result of frequent nasal obstruction caused by viral infection and inflammation that could limit the efficacy of noninvasive ventilation.  

The total complication rate was 9.9%. Surprisingly, they occurred mostly in failure trials instead of successfully-managed children who received ventilation support for more extended periods. Altered consciousness due to hypoxemia or hypercapnia, immobility and hemodynamic alterations in critically-ill children might be the underlying cause of this outcome. Skin/mucosa breakdown appeared to be the most common complication overall (4.8%), mainly observed at full-face masks. The incidence was within the reported range between 4% and 27% [4,39]. 

The study had several limitations. First of all, the scope of this research consisted of clinical observations on ventilation techniques and daily implementations. Thus we did not set any criteria for the decision of therapy institution or NIV failure. The call of intubation might have created subjectivity due to individual approaches of the collaborators. Secondly, we did not gather information on the decision-making process of the attending physicians to make any assumptions (e.g., the underlying factors for low ARDS percentage or the absence in EPAP/PEEP change throughout the ventilation session). The data on sedation sessions were also limited; whether if they were based on sedation protocols at each participating PICU or not. Despite the limitations, the prospective, observational, multi-centered nature of the study enabled us to observe the daily practice of NIV implementations and interpret a wide range of variations at multiple PICUs. The study also allowed us to improve our knowledge of ventilation techniques and to design local protocols to identify the ones at risk for developing respiratory failure. Furthermore, NIV failure was defined as intubation or re-intubation within 48 h [6]. Five days of study period has given the opportunity to observe whether the decision of failure has extended over 48 h or not. Only 4 children have failed their ventilation sessions after 48 h, therefore the statistics have included the first two days of the ventilation sessions.

## 5. Conclusion 

Age, acute disease, length of previous intubation, and the degree of hypoxemia adjudicated the selection of therapy modality. The failure rates were 17.6 and 11% at NIPPV and HFNC deliveries. A less than 10% decline in respiration within an hour, FiO2 requirement >55% at the 6th hour, and a PRISM-3 score >8 were three independent risk factors related to NIV failure. Instead of patient demographics (age, sex, and nature of respiratory failure), therapy modality (NIPPV or HFNC) and the institution of NIV as a first-line or rescue therapy, the nature of the acute disease (sepsis and ARDS), prolonged intubation prior to NIV, and the use of nasal masks were associated with therapy.

## Informed consent

The study protocol was approved by Akdeniz University Faculty of Medicine, Clinical Research Ethics Committee (no: 70904504/525, date: 11.18.2016) and followed the principles for human investigations outlined in the Second Declaration of Helsinki. It was also registered at clinicaltrials.gov (approval number: 70904504/525).
